# Characterization of the complete mitochondrial genome and phylogenetic relationship of *Pomatorhinus ruficollis* (Passeriformes, Timaliinae)

**DOI:** 10.1080/23802359.2016.1144100

**Published:** 2016-03-28

**Authors:** Qin Zhao, Huai-liang Xu, Bo Li, Meng Xie, Di-yan Li, Qing-yong Ni, Ming-wang Zhang, Yong-fang Yao

**Affiliations:** a College of Animal Science and Technology, Sichuan Agricultural University, Chengdu, Sichuan, China;; b College of Life Science, Sichuan Agricultural University, Ya’an, Sichuan, China

**Keywords:** Mitochondrial genome, Pomatorhinus, *Pomatorhinus ruficollis*

## Abstract

In our study, we first report complete mitogenome for *P. ruficollis* and obtain basic genetic information. The genome of *P. ruficollis* is 17 009 bp which contained 13 protein-coding genes, 22 transfer RNA genes, two ribosomal RNA genes and two control regions. Overall bases composition of the complete mitochondrial genome is 29.70%A, 14.47%G, 23.31% T, 32.52%C. Twelve PCGs and 14 tRNA genes are distributed on the H-strand, *ND6* and eight tRNA genes are encoded on the L-strand.

*Pomatorhinus ruficollis* (Rufous-necked scimitar babbler) is one of the members in *Pomatorhinus* genus, they distributed in northern Indochina, northern and western Burma, central to southern China and sino-Himalayan. *Pomatorhinus rufillos* are mainly live in lowlands, foothills and montane forests (Nyari & Reddy 2013) range altitude from 200 m to 3000 m (Song et al. 2011). The population of *P. ruficollis* sudden drop due to e-waste polluted (Zhang et al. 2015). Because *P. ruficollis* chest differ in the colours spots, vertical lines and it can be slipted into at least three groups (Cheng 1964; Collar 2006; Collar & Robson 2007). Based on the morphological characteristics of *P. rufillos* includes 14 subspecies (Cheng 1962). *P.r. musicus* is promoted to be independent specie with morphometric differences (Collar 2004, 2006). Therefore, *P. ruficollis* contains 13 subspecies (Song et al. 2011). The complete mitochondrial genome of *P.ruficollis* will be helpful for the deeply study on the genetic phylogeny and can provided the method for species conservation.

In our experiment, the muscle sample of *P. ruficollis* was collected from Ya'an (N30°01', E103°02') and stored at the Wildlife Conservation Laboratory, Sichuan Agricultural University, Sichuan province, China. The mitochondrial genome extracted from the muscle tissue by phenol-chloroform method (Sambrook & Russell 2001). We designed 21 pairs of normal PCR primers and four pairs LA-PCR primers by primer 5.0 according to complete mitochondrial genome of *Garrulax cineraceus* (GeneBank accession no. NC024553). MAGE6.0 (Tamura et al., 2013) is used to construct neighbour-joining (NJ) tree.

The complete mitogenome of *P. ruficollis* is 17 009 bp submitted to GenBank (KT970675). The mitogenome have 13 protein-coding genes (PCGs), 22 tRNA genes, two rRNA genes and two control regions (D-loop). Twelve PCGs and 14 tRNA are distributed on the H-strand, *ND6* and eight tRNA genes are encoded on the L-strand. The base composition of mitogenome is 29.70%A, 14.47%G, 23.31% T and 32.52%C, so the percentage of A + T (53.01%) is slightly higher than G + C (46.99%). Twelve of the 13 protein-coding genes regard ATG as start codon, except GTG for *ND1* gene. Four PCGs (*ND2, COX3, ND3* and *ND4*) showed incomplete stop codons (T or TA), the five genes (*COX2*, *ATP8*, *ATP6*, *ND4* and *Cytb*) end with TAA, *ND1* and *ND5* stops with AGA, *COX1* use AGG, and *ND6* terminated with TGA. Twelve SrRNA and 16 SrRNA are located between *tRNA^Phe^* and *tRNA^Leu^(UUR)* that is separated by *tRNA^Val^*, with the size of 982 and 1601 bp, respectively. The two control regions (CR) of the *P. ruficollis* are 1058 bp and 385 bp, *CR1* is located between the *tRNA^Thr^* and *tRNA^Pro^*and *CR2* between *tRNA^Glu^*and tRNA^Phe^. *Pomatorhinus ruficollis* complete mitochondrial genome structure is similar to the typical mitochondrial genomse of vertebrates (Qian et al. 2012; Zhang et al. 2013; Duan et al. 2014).

Figure 1 shows that *Pomatorhinus* genus gathered to one clade, *Xiphirhynchus superciliaris* and *Pomatorhinus ferruginosus* are clustered as a sister lineage. *Pomatorhinus* is not monophyly, which is consistent with other studies (Cibois 2003; Collar 2004; Reddy & Moyle 2010; Moyle et al. 2012).

**Figure 1.**
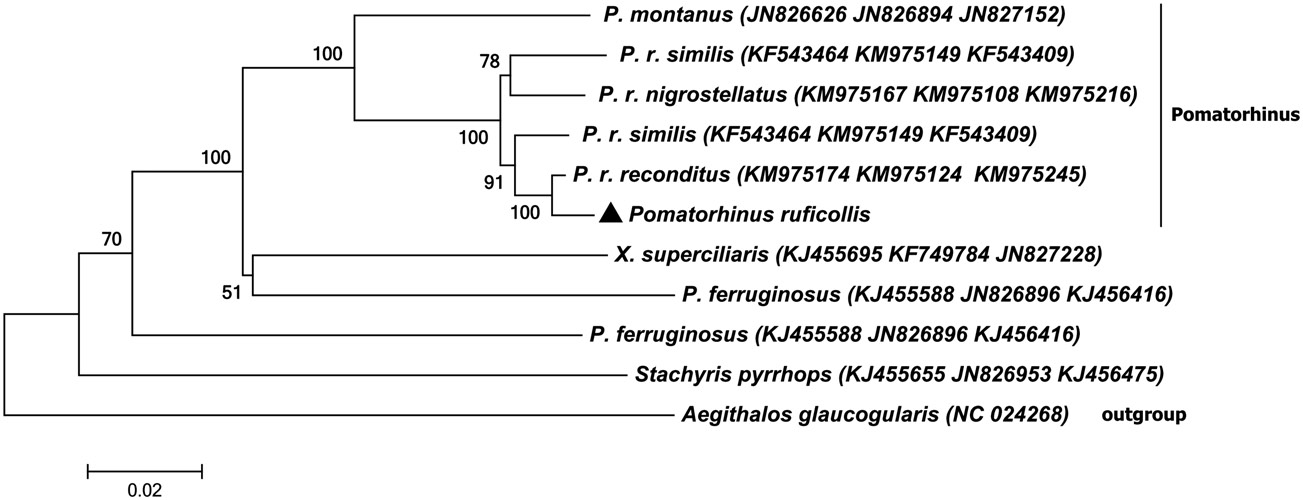
The phylogenetic tree used the method of neighbour-joining (NJ) tree based on *ND2, ND3, Cytb* protein-coding genes with Kimura 2-parameter model by using MEGA 6.0 program (MEGA Inc., Englewood, NJ). The number on each branch indicated the local bootstrap value. The *ND2, ND3* and *Cytb* Genebank accession number are arranged in brackets after the name of the species.
